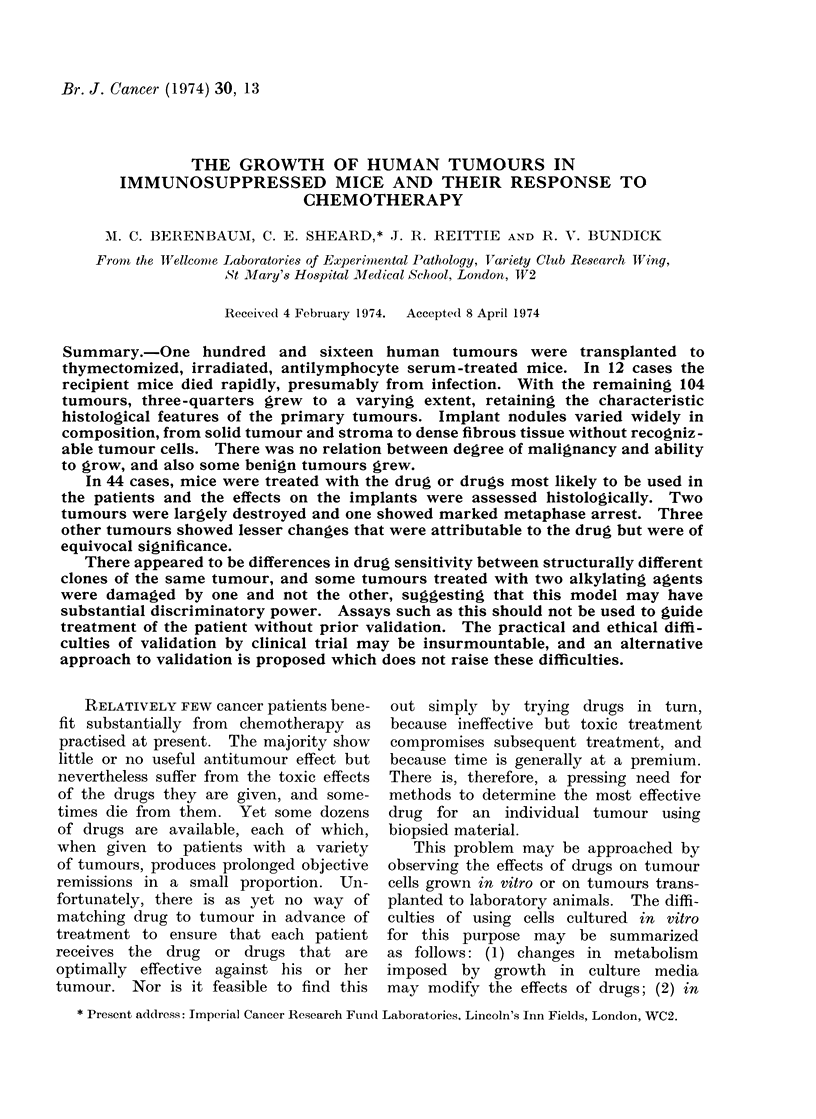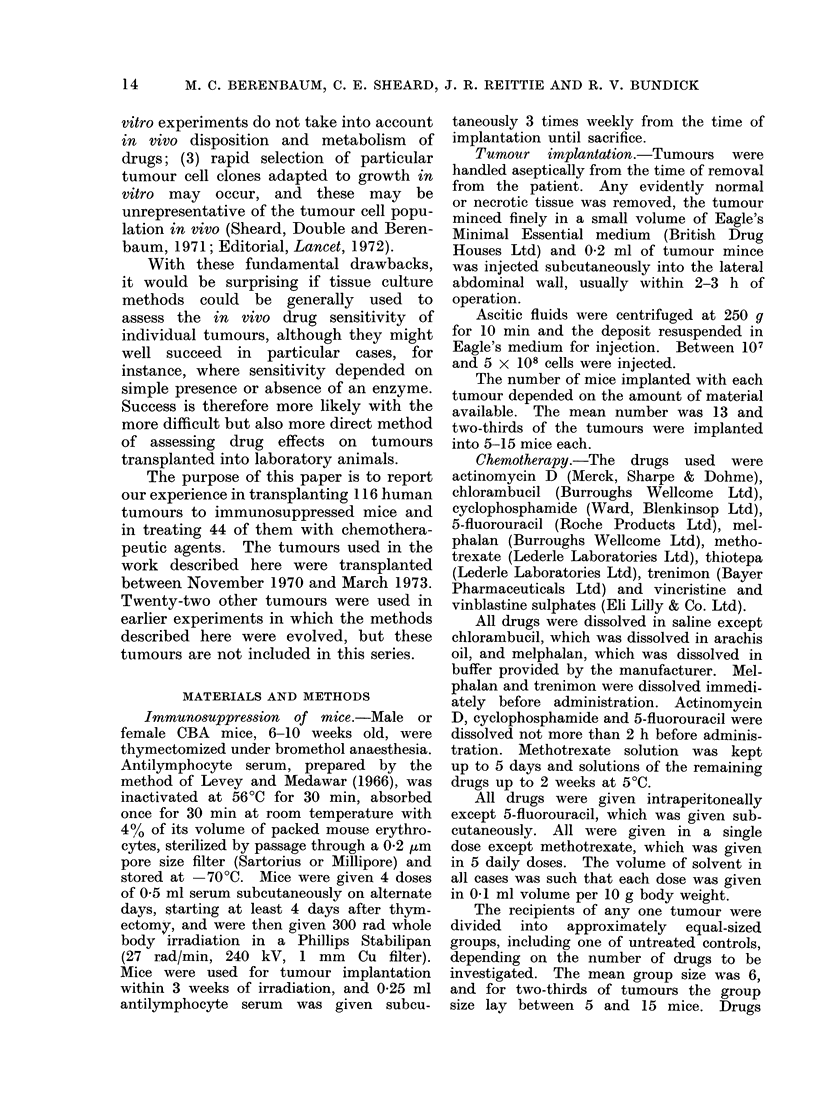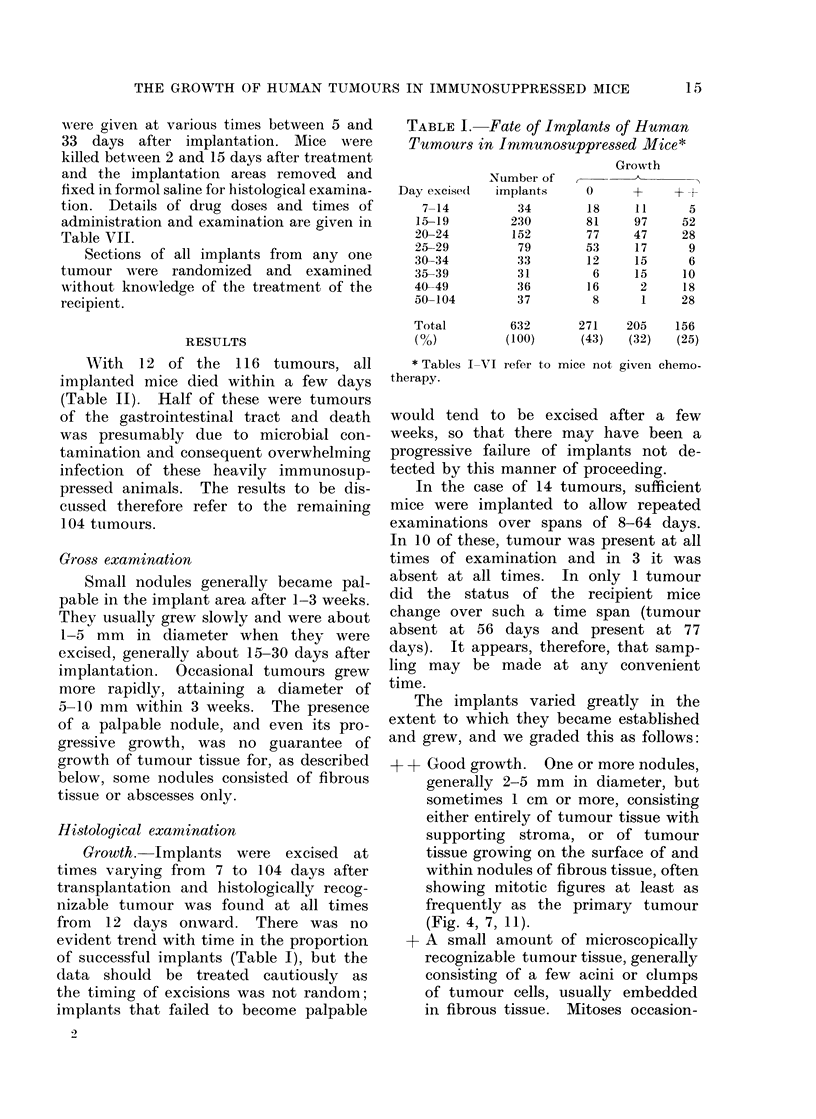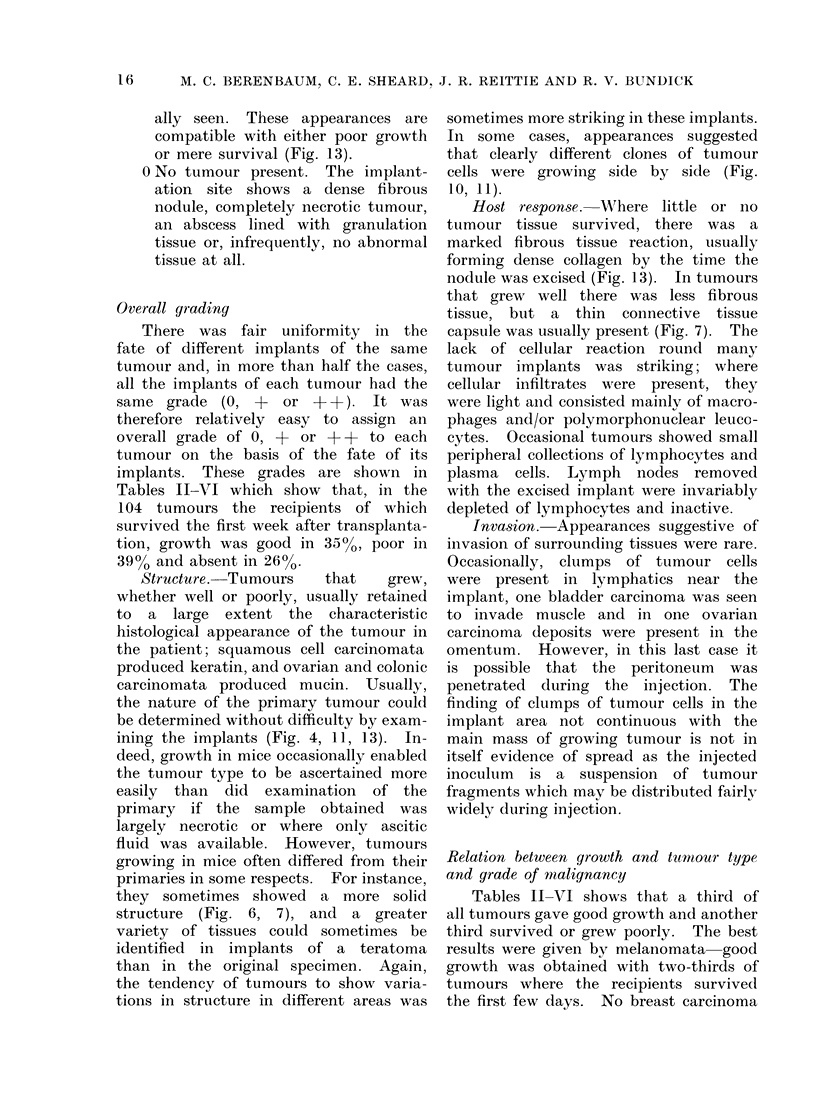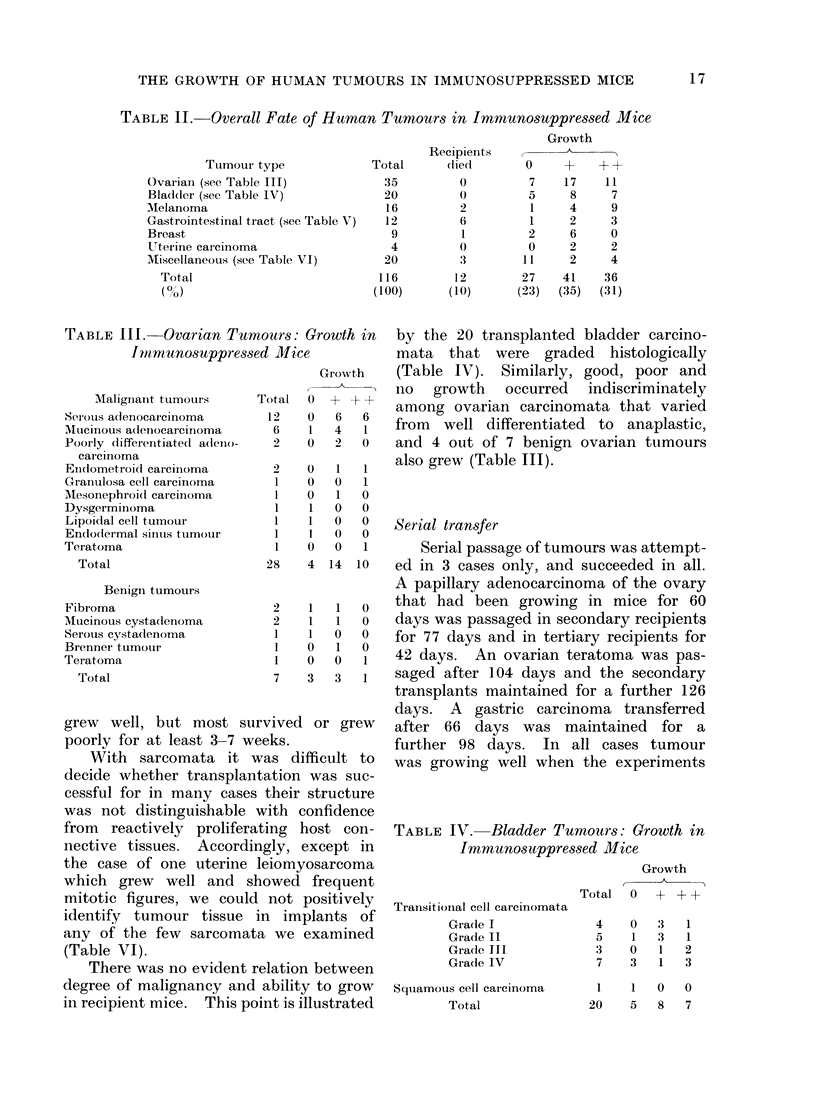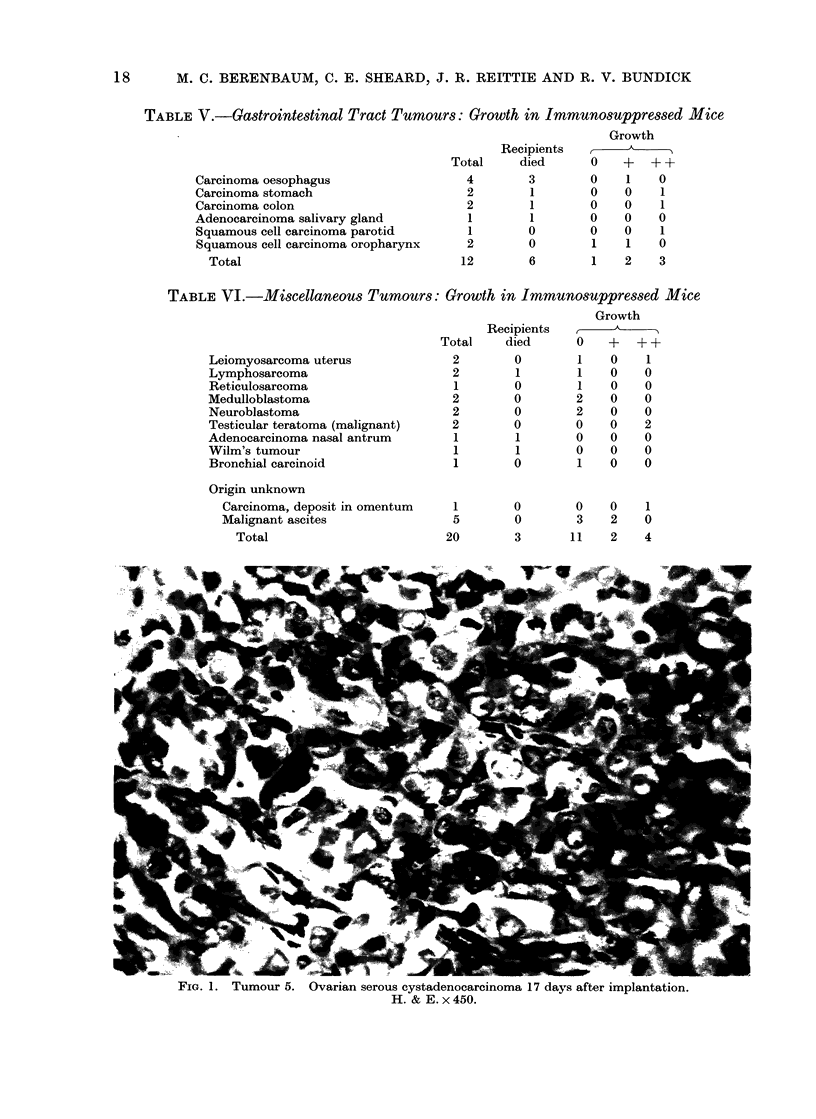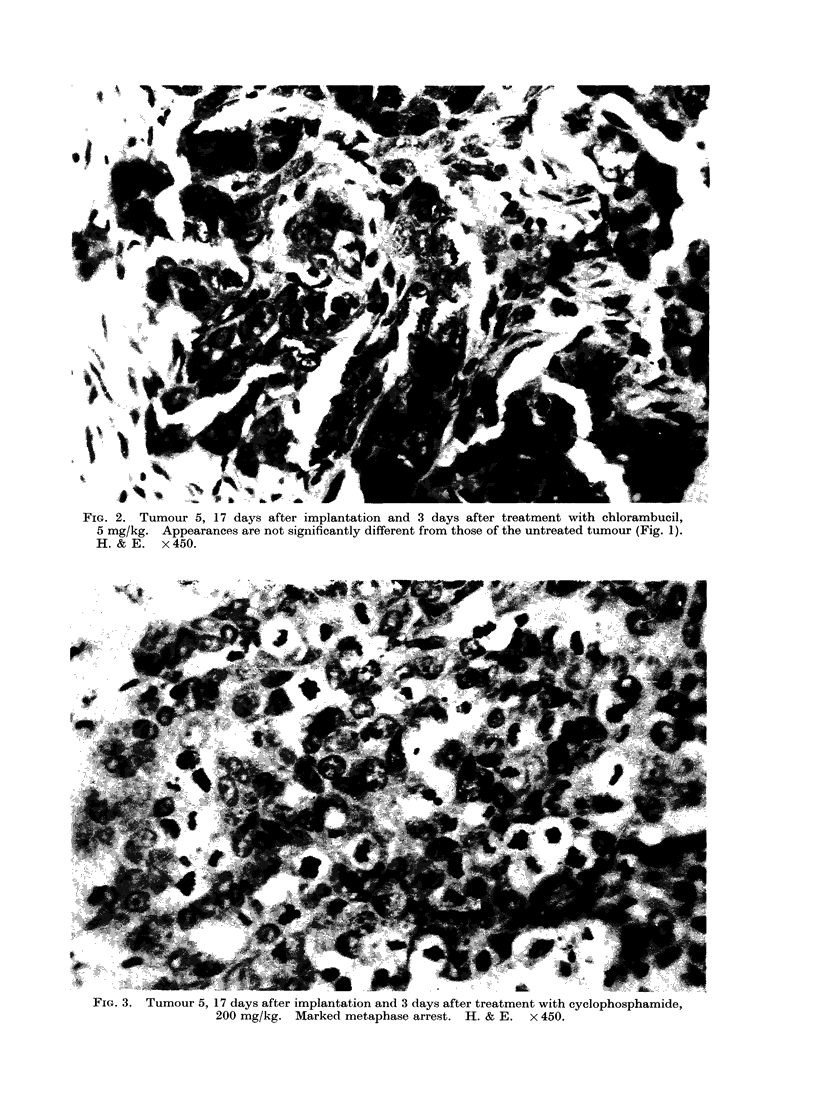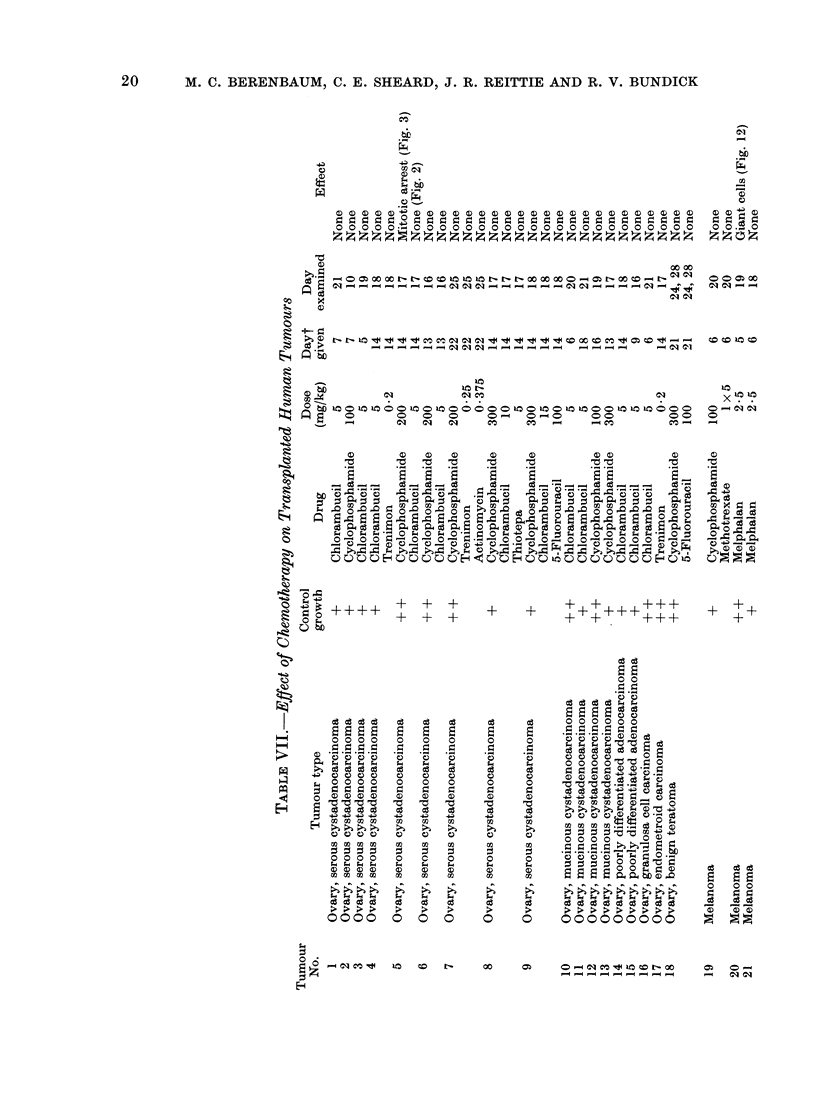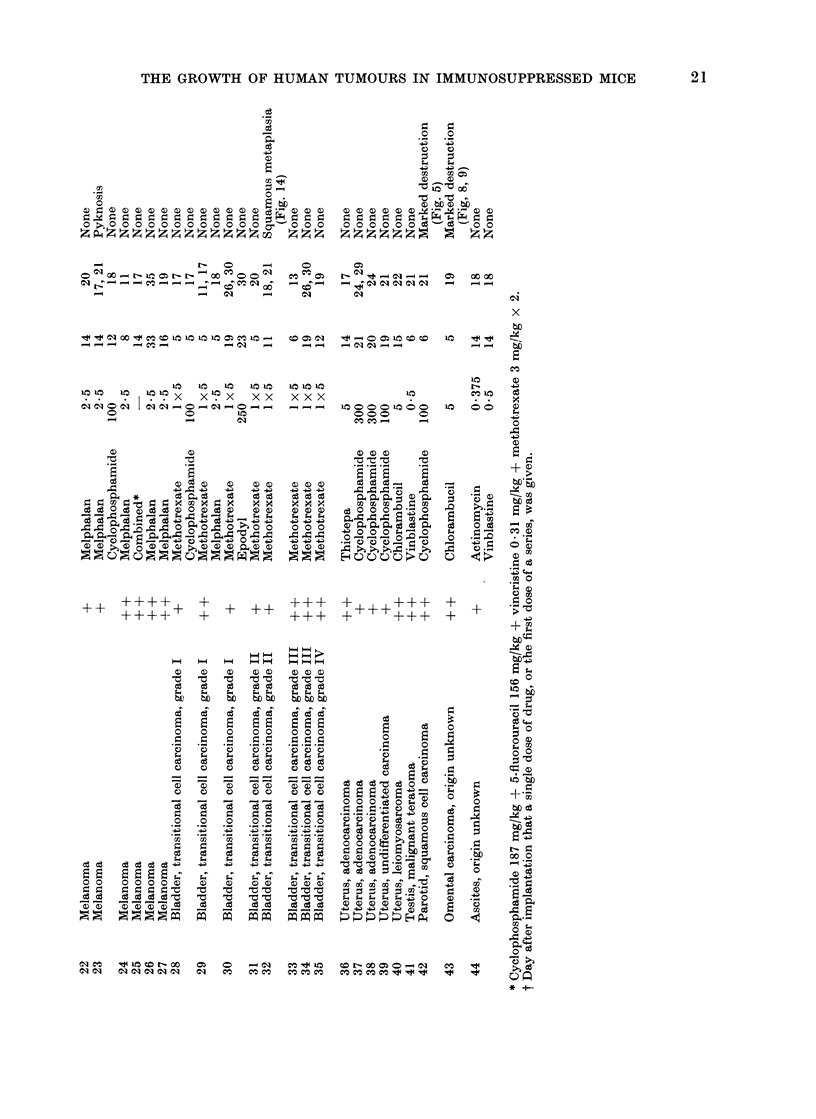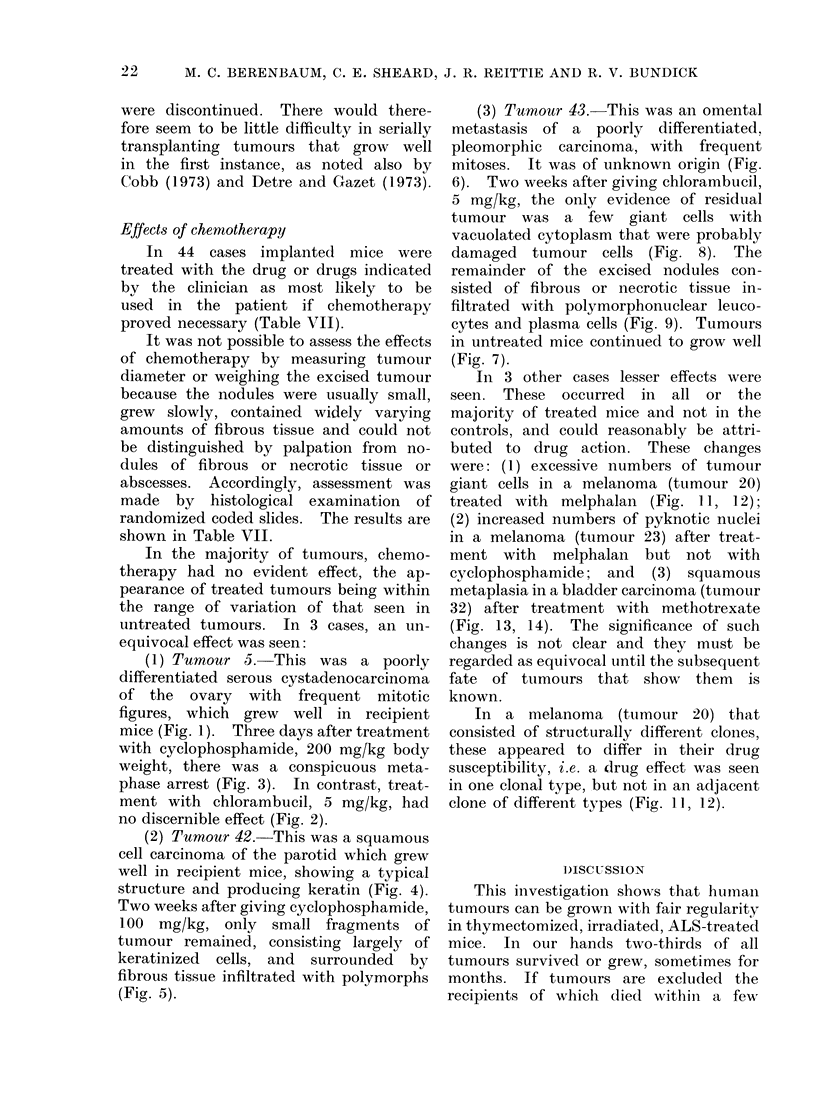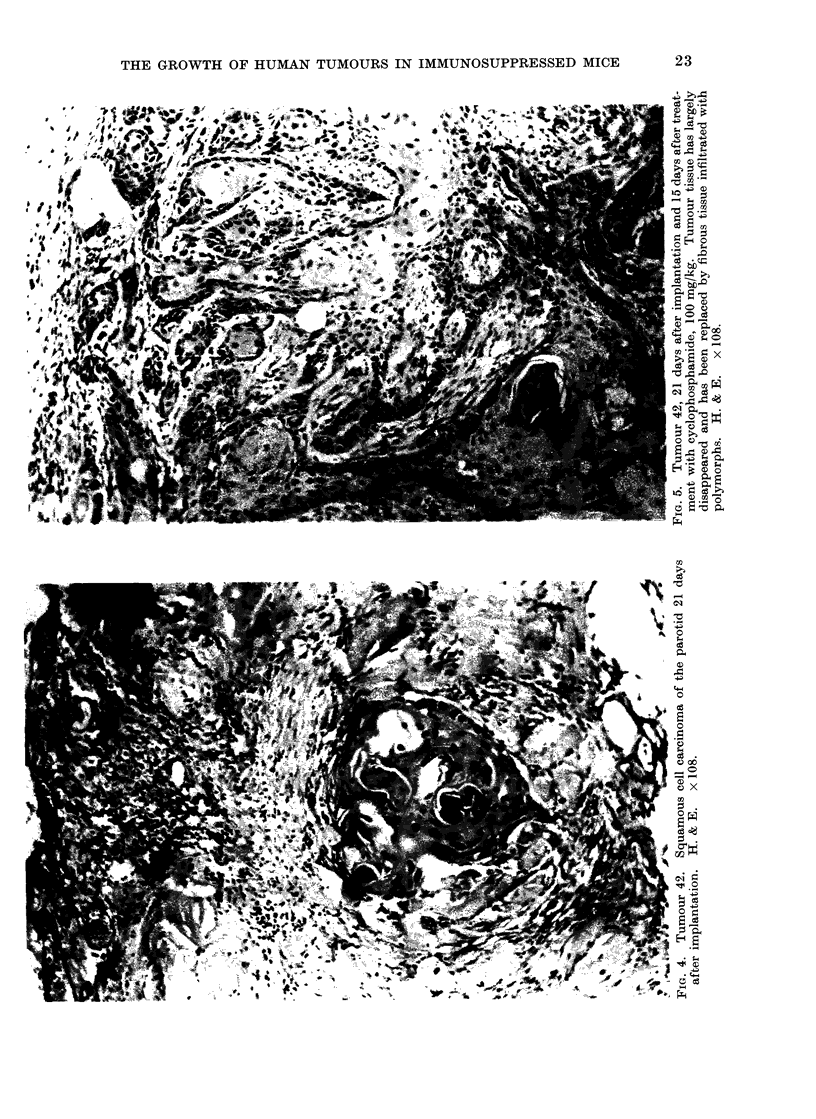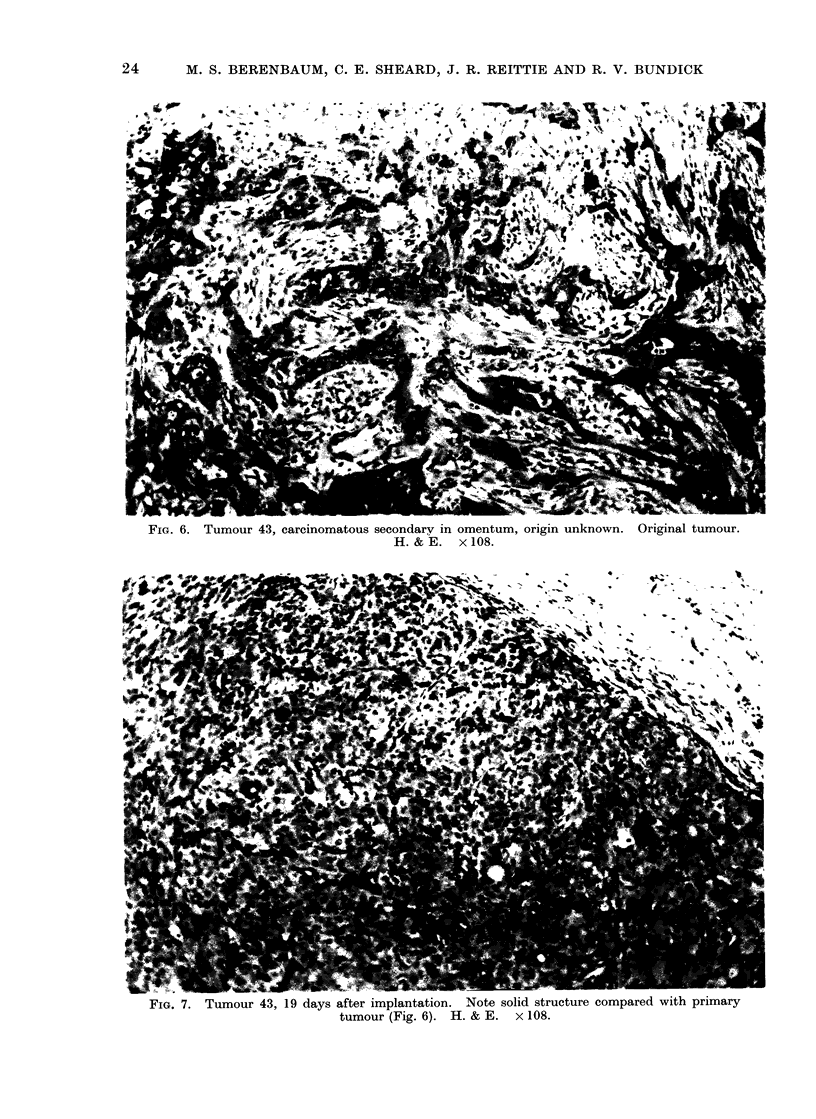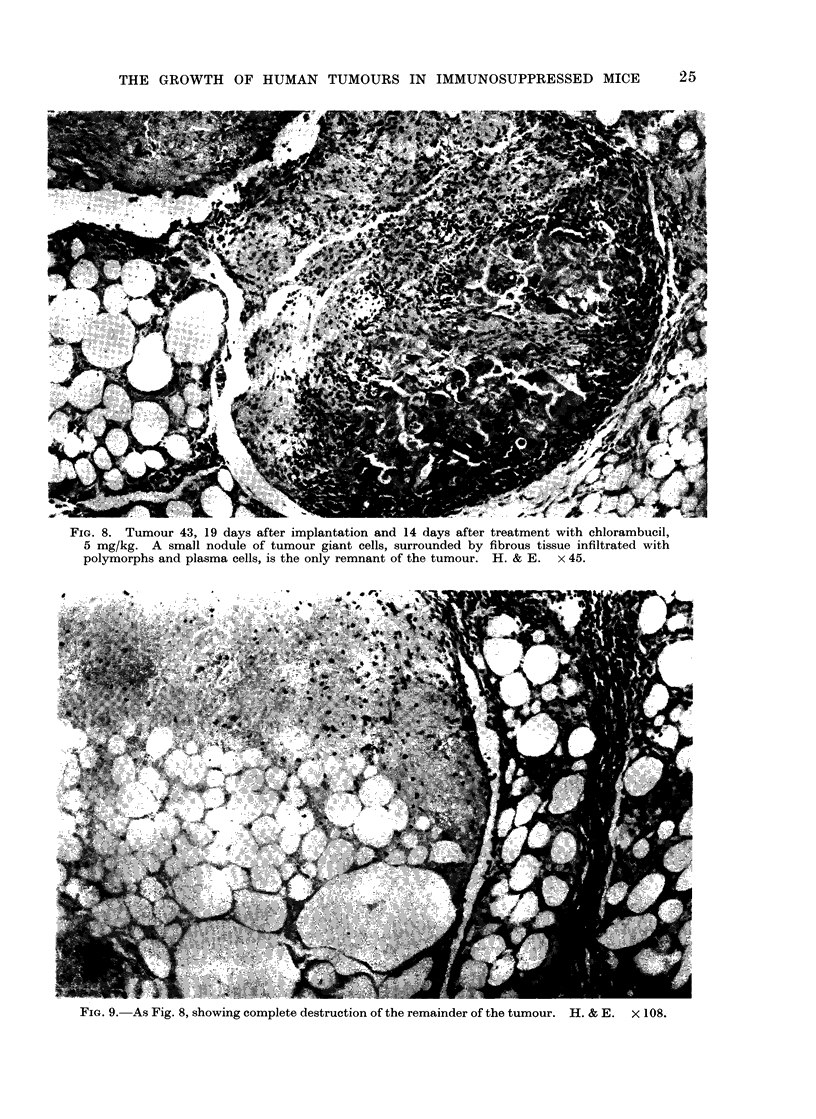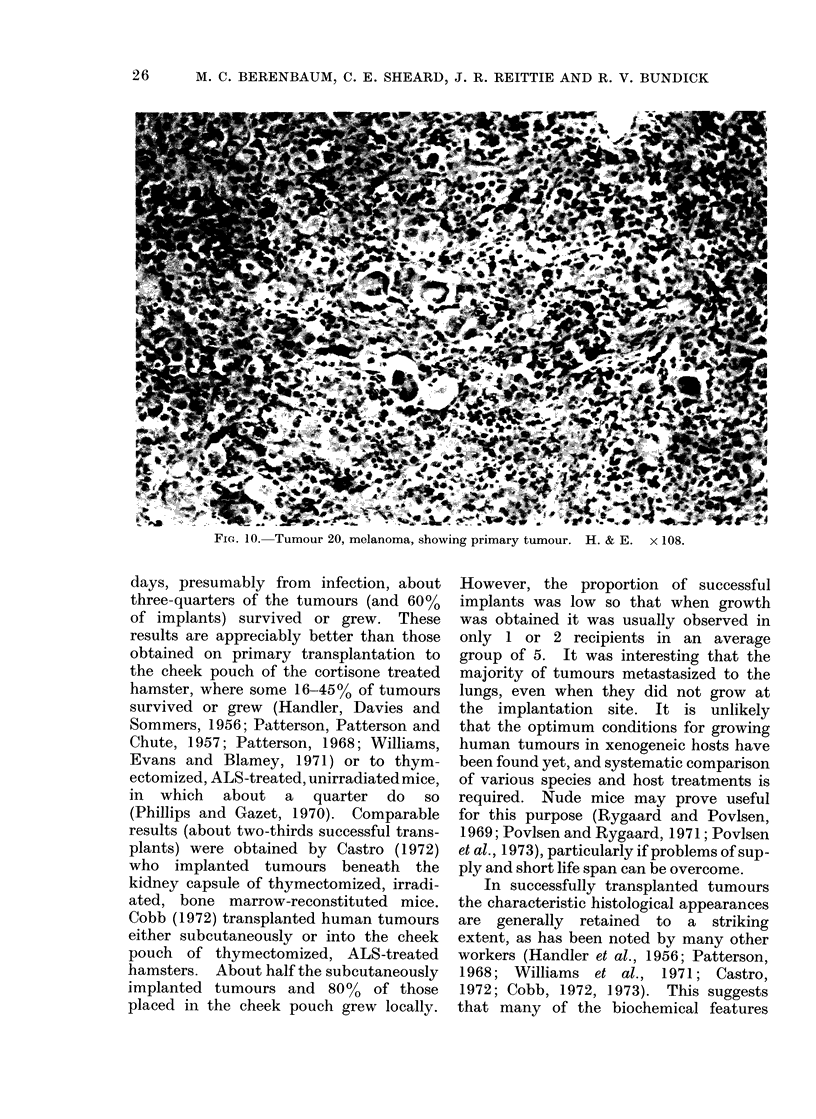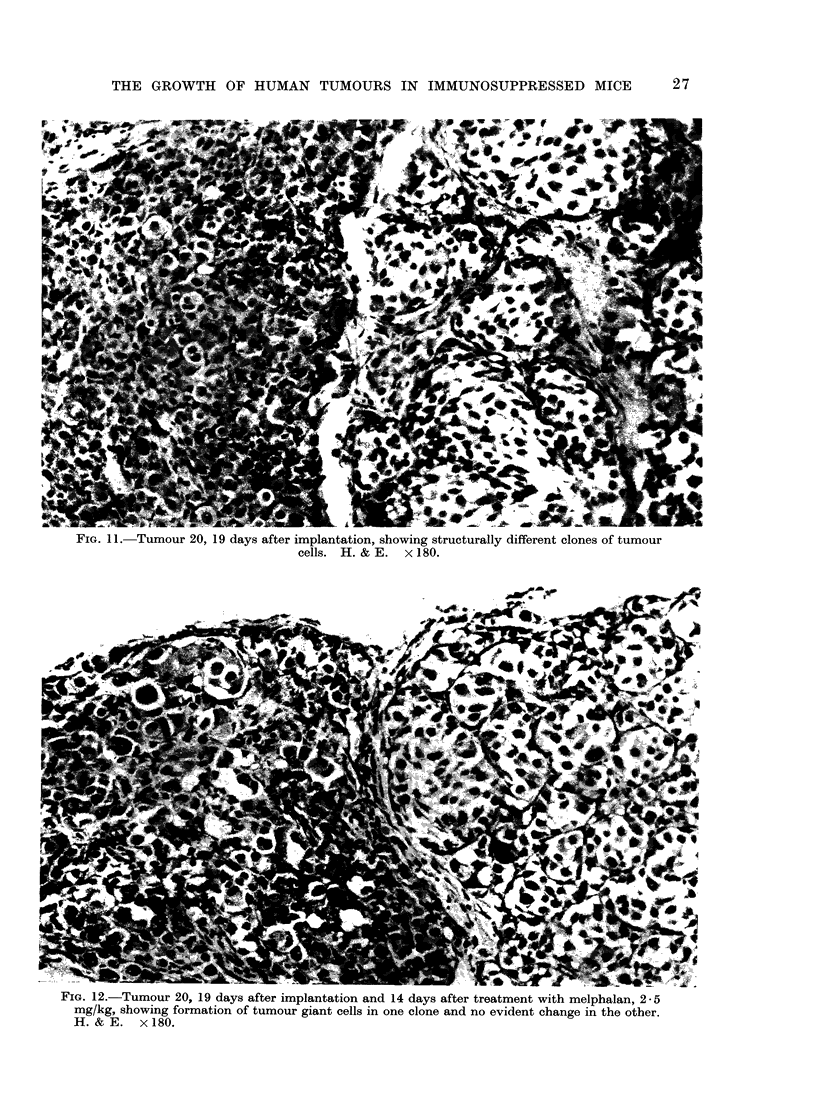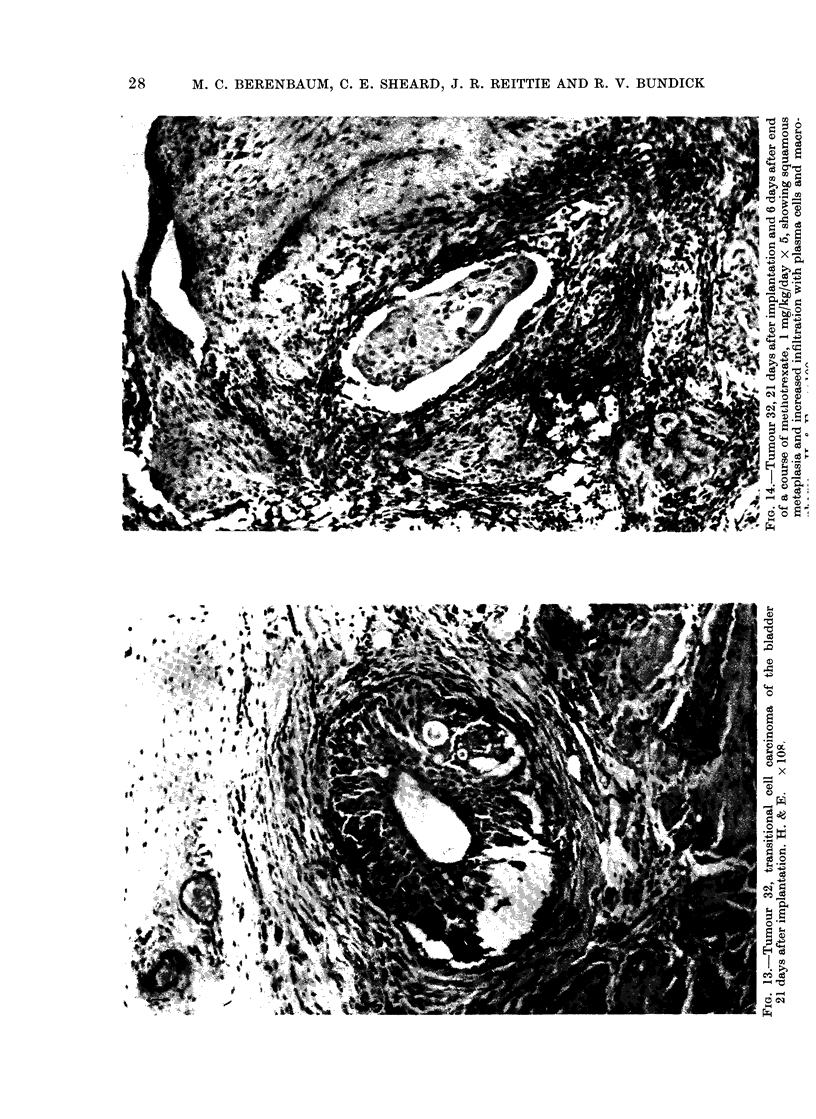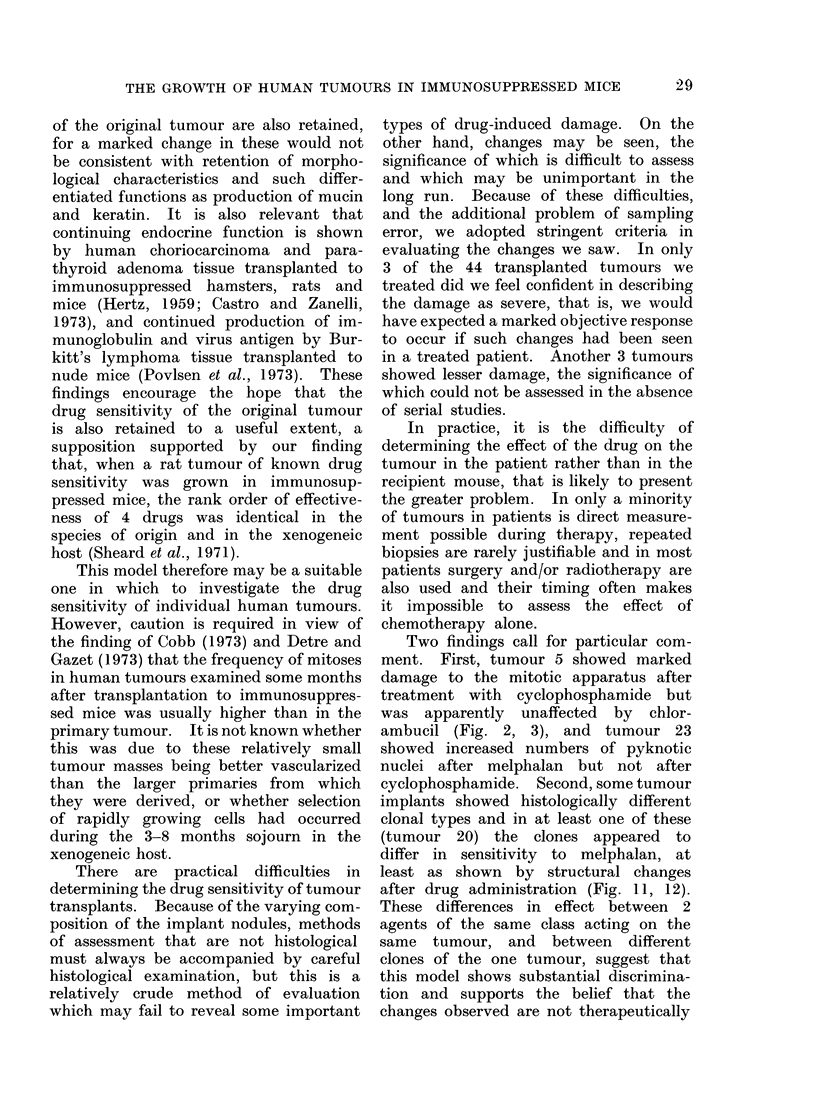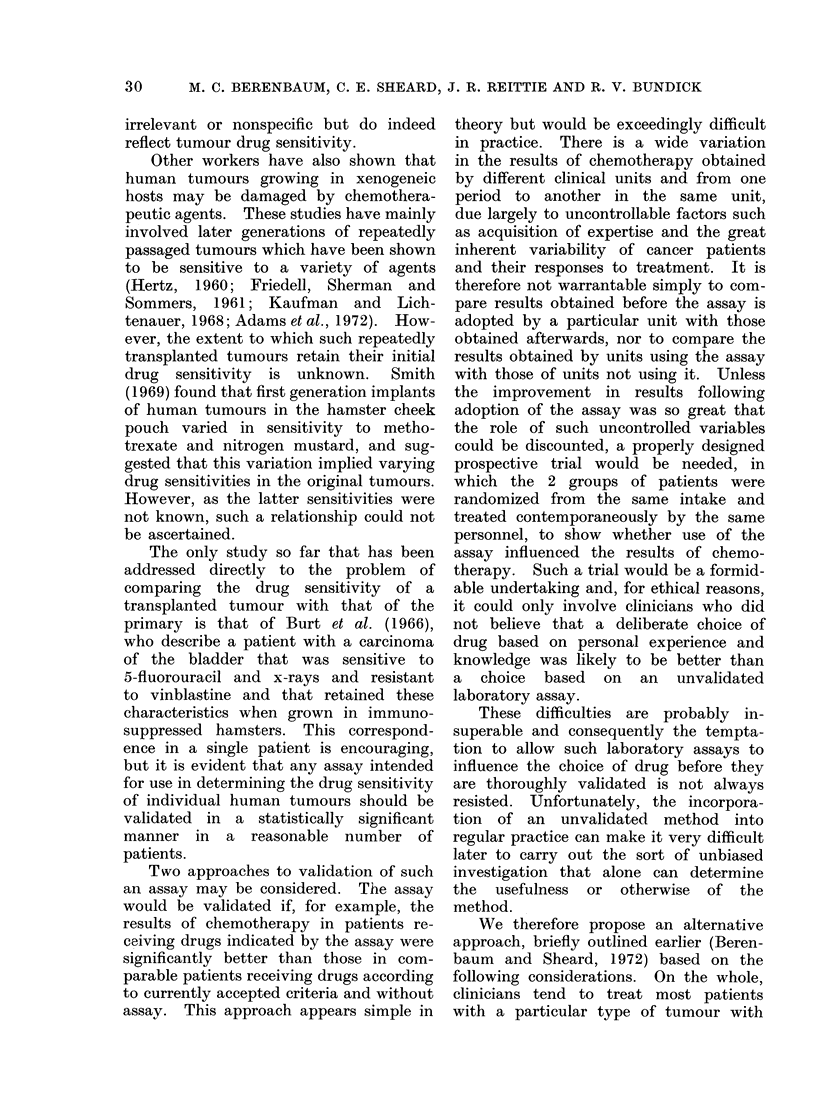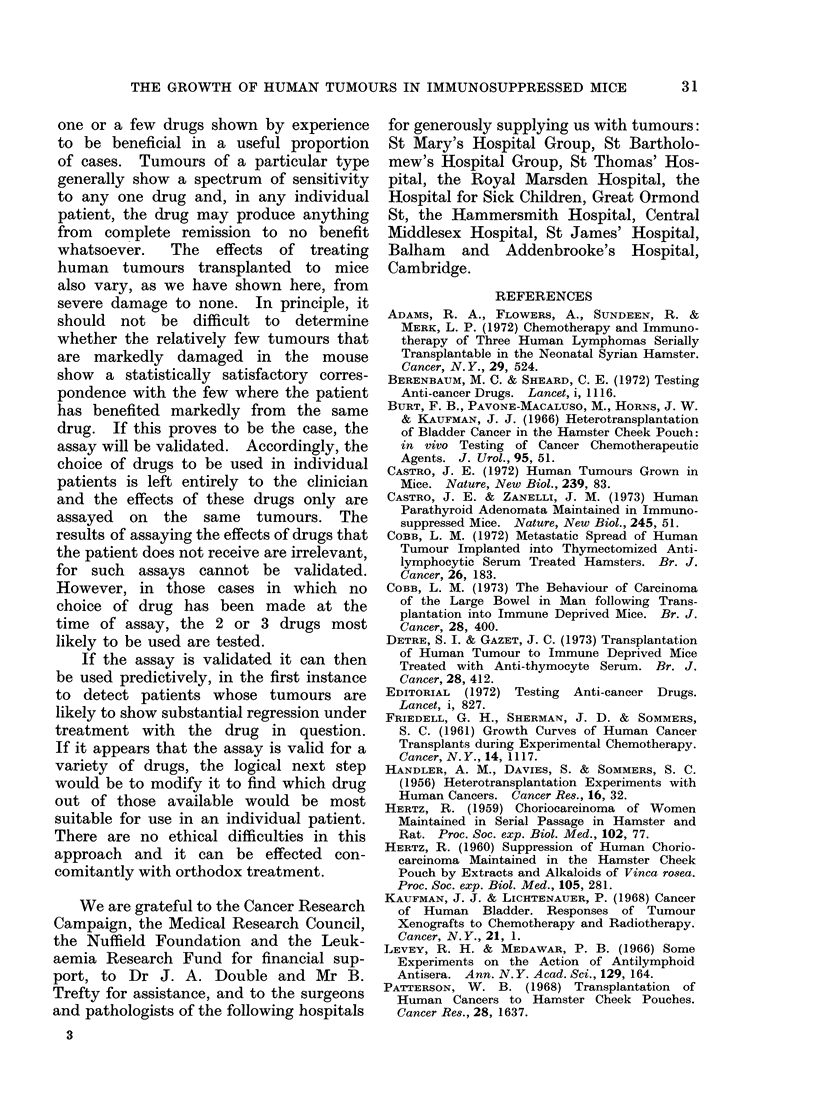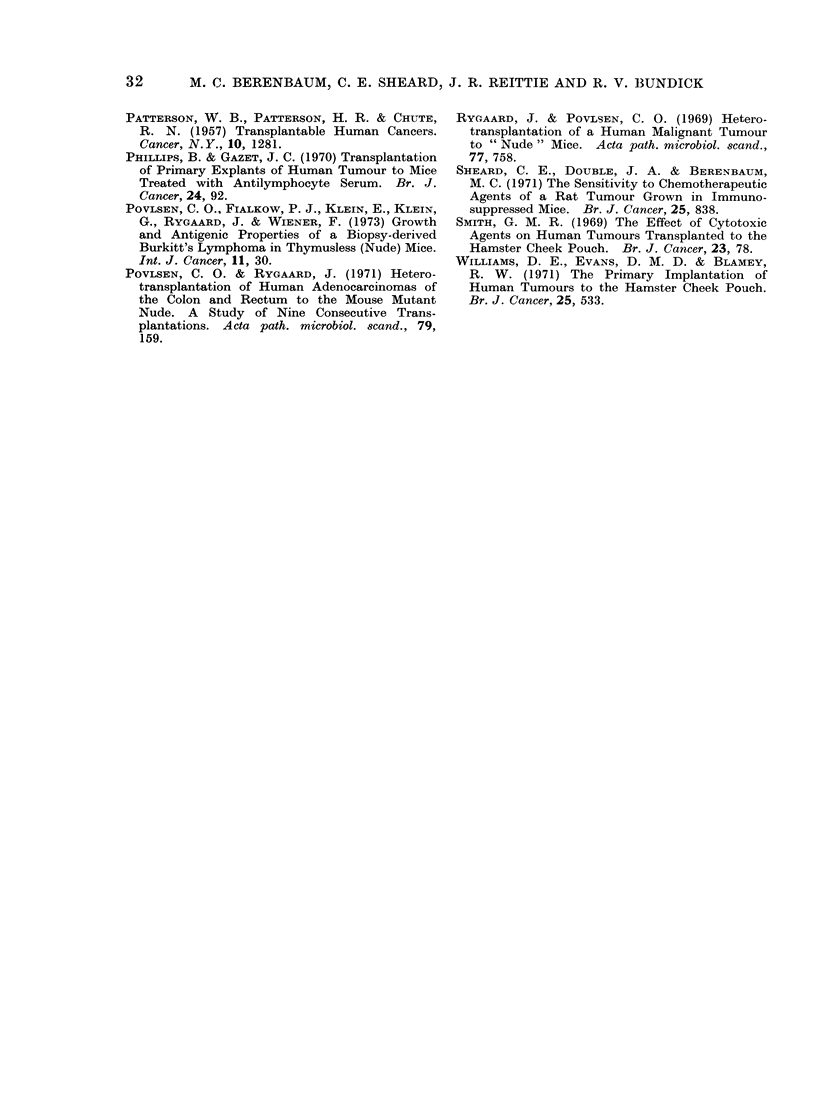# The Growth of Human Tumours in Immunosuppressed Mice and Their Response to Chemotherapy

**DOI:** 10.1038/bjc.1974.109

**Published:** 1974-07

**Authors:** M. C. Berenbaum, C. E. Sheard, J. R. Reittie, R. V. Bundick

## Abstract

**Images:**


Br. J. Cancer (1974) 30, 13

### THE GROWTH OF HUMAN TUMOURS IN IMMUNOSUPPRESSED MICE AND THEIR RESPONSE TO CHEMOTHERAPY

M. C. BERENBAUM, C. E. SHEARD,* J. R. REITTIE AND R. V. BUNDICK

*From the Wellcome Laboratories of Experimental Pathology, Variety Club Research Wing, St Mary's Hospital Medical School, London, W2*

Received 4 February 1974. Accepted 8 April 1974

**Summary.—One hundred and sixteen human tumours were transplanted to thymectomized, irradiated, antilymphocyte serum-treated mice. In 12 cases the recipient mice died rapidly, presumably from infection. With the remaining 104 tumours, three-quarters grew to a varying extent, retaining the characteristic histological features of the primary tumours. Implant nodules varied widely in composition, from solid tumour and stroma to dense fibrous tissue without recognizable tumour cells. There was no relation between degree of malignancy and ability to grow, and also some benign tumours grew.**

**In 44 cases, mice were treated with the drug or drugs most likely to be used in the patients and the effects on the implants were assessed histologically. Two tumours were largely destroyed and one showed marked metaphase arrest. Three other tumours showed lesser changes that were attributable to the drug but were of equivocal significance.**

**There appeared to be differences in drug sensitivity between structurally different clones of the same tumour, and some tumours treated with two alkylating agents were damaged by one and not the other, suggesting that this model may have substantial discriminatory power. Assays such as this should not be used to guide treatment of the patient without prior validation. The practical and ethical difficulties of validation by clinical trial may be insurmountable, and an alternative approach to validation is proposed which does not raise these difficulties.**

RELATIVELY FEW cancer patients benefit substantially from chemotherapy as practised at present. The majority show little or no useful antitumour effect but nevertheless suffer from the toxic effects of the drugs they are given, and sometimes die from them. Yet some dozens of drugs are available, each of which, when given to patients with a variety of tumours, produces prolonged objective remissions in a small proportion. Unfortunately, there is as yet no way of matching drug to tumour in advance of treatment to ensure that each patient receives the drug or drugs that are optimally effective against his or her tumour. Nor is it feasible to find this out simply by trying drugs in turn, because ineffective but toxic treatment compromises subsequent treatment, and because time is generally at a premium. There is, therefore, a pressing need for methods to determine the most effective drug for an individual tumour using biopsied material.

This problem may be approached by observing the effects of drugs on tumour cells grown *in vitro* or on tumours transplanted to laboratory animals. The difficulties of using cells cultured *in vitro* for this purpose may be summarized as follows: (1) changes in metabolism imposed by growth in culture media may modify the effects of drugs; (2) *in*

* Present address: Imperial Cancer Research Fund Laboratories, Lincoln's Inn Fields, London, WC2.

*vitro* experiments do not take into account *in vivo* disposition and metabolism of drugs; (3) rapid selection of particular tumour cell clones adapted to growth *in vitro* may occur, and these may be unrepresentative of the tumour cell population *in vivo* (Sheard, Double and Berenbaum, 1971; Editorial, *Lancet*, 1972).

With these fundamental drawbacks, it would be surprising if tissue culture methods could be generally used to assess the *in vivo* drug sensitivity of individual tumours, although they might well succeed in particular cases, for instance, where sensitivity depended on simple presence or absence of an enzyme. Success is therefore more likely with the more difficult but also more direct method of assessing drug effects on tumours transplanted into laboratory animals.

The purpose of this paper is to report our experience in transplanting 116 human tumours to immunosuppressed mice and in treating 44 of them with chemotherapeutic agents. The tumours used in the work described here were transplanted between November 1970 and March 1973. Twenty-two other tumours were used in earlier experiments in which the methods described here were evolved, but these tumours are not included in this series.

### MATERIALS AND METHODS

*Immunosuppression of mice.*—Male or female CBA mice, 6–10 weeks old, were thymectomized under bromethol anaesthesia. Antilymphocyte serum, prepared by the method of Levey and Medawar (1966), was inactivated at 56°C for 30 min, absorbed once for 30 min at room temperature with 4% of its volume of packed mouse erythrocytes, sterilized by passage through a 0·2 $\mu$m pore size filter (Sartorius or Millipore) and stored at −70°C. Mice were given 4 doses of 0·5 ml serum subcutaneously on alternate days, starting at least 4 days after thymectomy, and were then given 300 rad whole body irradiation in a Phillips Stabilipan (27 rad/min, 240 kV, 1 mm Cu filter). Mice were used for tumour implantation within 3 weeks of irradiation, and 0·25 ml antilymphocyte serum was given subcutaneously 3 times weekly from the time of implantation until sacrifice.

*Tumour implantation.*—Tumours were handled aseptically from the time of removal from the patient. Any evidently normal or necrotic tissue was removed, the tumour minced finely in a small volume of Eagle's Minimal Essential medium (British Drug Houses Ltd) and 0·2 ml of tumour mince was injected subcutaneously into the lateral abdominal wall, usually within 2–3 h of operation.

Ascitic fluids were centrifuged at 250 *g* for 10 min and the deposit resuspended in Eagle's medium for injection. Between $10^7$ and $5 \times 10^8$ cells were injected.

The number of mice implanted with each tumour depended on the amount of material available. The mean number was 13 and two-thirds of the tumours were implanted into 5–15 mice each.

*Chemotherapy.*—The drugs used were actinomycin D (Merck, Sharpe & Dohme), chlorambucil (Burroughs Wellcome Ltd), cyclophosphamide (Ward, Blenkinsop Ltd), 5-fluorouracil (Roche Products Ltd), melphalan (Burroughs Wellcome Ltd), methotrexate (Lederle Laboratories Ltd), thiotepa (Lederle Laboratories Ltd), trenimon (Bayer Pharmaceuticals Ltd) and vincristine and vinblastine sulphates (Eli Lilly & Co. Ltd).

All drugs were dissolved in saline except chlorambucil, which was dissolved in arachis oil, and melphalan, which was dissolved in buffer provided by the manufacturer. Melphalan and trenimon were dissolved immediately before administration. Actinomycin D, cyclophosphamide and 5-fluorouracil were dissolved not more than 2 h before administration. Methotrexate solution was kept up to 5 days and solutions of the remaining drugs up to 2 weeks at 5°C.

All drugs were given intraperitoneally except 5-fluorouracil, which was given subcutaneously. All were given in a single dose except methotrexate, which was given in 5 daily doses. The volume of solvent in all cases was such that each dose was given in 0·1 ml volume per 10 g body weight.

The recipients of any one tumour were divided into approximately equal-sized groups, including one of untreated controls, depending on the number of drugs to be investigated. The mean group size was 6, and for two-thirds of tumours the group size lay between 5 and 15 mice. Drugs were given at various times between 5 and 33 days after implantation. Mice were killed between 2 and 15 days after treatment and the implantation areas removed and fixed in formol saline for histological examination. Details of drug doses and times of administration and examination are given in Table VII.

Sections of all implants from any one tumour were randomized and examined without knowledge of the treatment of the recipient.

TABLE I.—*Fate of Implants of Human Tumours in Immunosuppressed Mice**

| Day excised | Number of implants | Growth 0 | + | ++ |
|---|---|---|---|---|
| 7–14 | 34 | 18 | 11 | 5 |
| 15–19 | 230 | 81 | 97 | 52 |
| 20–24 | 152 | 77 | 47 | 28 |
| 25–29 | 79 | 53 | 17 | 9 |
| 30–34 | 33 | 12 | 15 | 6 |
| 35–39 | 31 | 6 | 15 | 10 |
| 40–49 | 36 | 16 | 2 | 18 |
| 50–104 | 37 | 8 | 1 | 28 |
| Total | 632 | 271 | 205 | 156 |
| (%) | (100) | (43) | (32) | (25) |

* Tables I–VI refer to mice not given chemotherapy.

### RESULTS

With 12 of the 116 tumours, all implanted mice died within a few days (Table II). Half of these were tumours of the gastrointestinal tract and death was presumably due to microbial contamination and consequent overwhelming infection of these heavily immunosuppressed animals. The results to be discussed therefore refer to the remaining 104 tumours.

### *Gross examination*

Small nodules generally became palpable in the implant area after 1–3 weeks. They usually grew slowly and were about 1–5 mm in diameter when they were excised, generally about 15–30 days after implantation. Occasional tumours grew more rapidly, attaining a diameter of 5–10 mm within 3 weeks. The presence of a palpable nodule, and even its progressive growth, was no guarantee of growth of tumour tissue for, as described below, some nodules consisted of fibrous tissue or abscesses only.

### *Histological examination*

*Growth.*—Implants were excised at times varying from 7 to 104 days after transplantation and histologically recognizable tumour was found at all times from 12 days onward. There was no evident trend with time in the proportion of successful implants (Table I), but the data should be treated cautiously as the timing of excisions was not random; implants that failed to become palpable would tend to be excised after a few weeks, so that there may have been a progressive failure of implants not detected by this manner of proceeding.

In the case of 14 tumours, sufficient mice were implanted to allow repeated examinations over spans of 8–64 days. In 10 of these, tumour was present at all times of examination and in 3 it was absent at all times. In only 1 tumour did the status of the recipient mice change over such a time span (tumour absent at 56 days and present at 77 days). It appears, therefore, that sampling may be made at any convenient time.

The implants varied greatly in the extent to which they became established and grew, and we graded this as follows:

++ Good growth. One or more nodules, generally 2–5 mm in diameter, but sometimes 1 cm or more, consisting either entirely of tumour tissue with supporting stroma, or of tumour tissue growing on the surface of and within nodules of fibrous tissue, often showing mitotic figures at least as frequently as the primary tumour (Fig. 4, 7, 11).

\+ A small amount of microscopically recognizable tumour tissue, generally consisting of a few acini or clumps of tumour cells, usually embedded in fibrous tissue. Mitoses occasionally seen. These appearances are compatible with either poor growth or mere survival (Fig. 13).

0 No tumour present. The implantation site shows a dense fibrous nodule, completely necrotic tumour, an abscess lined with granulation tissue or, infrequently, no abnormal tissue at all.

*Overall grading*

There was fair uniformity in the fate of different implants of the same tumour and, in more than half the cases, all the implants of each tumour had the same grade (0, + or ++). It was therefore relatively easy to assign an overall grade of 0, + or ++ to each tumour on the basis of the fate of its implants. These grades are shown in Tables II–VI which show that, in the 104 tumours the recipients of which survived the first week after transplantation, growth was good in 35%, poor in 39% and absent in 26%.

*Structure.*—Tumours that grew, whether well or poorly, usually retained to a large extent the characteristic histological appearance of the tumour in the patient; squamous cell carcinomata produced keratin, and ovarian and colonic carcinomata produced mucin. Usually, the nature of the primary tumour could be determined without difficulty by examining the implants (Fig. 4, 11, 13). Indeed, growth in mice occasionally enabled the tumour type to be ascertained more easily than did examination of the primary if the sample obtained was largely necrotic or where only ascitic fluid was available. However, tumours growing in mice often differed from their primaries in some respects. For instance, they sometimes showed a more solid structure (Fig. 6, 7), and a greater variety of tissues could sometimes be identified in implants of a teratoma than in the original specimen. Again, the tendency of tumours to show variations in structure in different areas was sometimes more striking in these implants. In some cases, appearances suggested that clearly different clones of tumour cells were growing side by side (Fig. 10, 11).

*Host response.*—Where little or no tumour tissue survived, there was a marked fibrous tissue reaction, usually forming dense collagen by the time the nodule was excised (Fig. 13). In tumours that grew well there was less fibrous tissue, but a thin connective tissue capsule was usually present (Fig. 7). The lack of cellular reaction round many tumour implants was striking; where cellular infiltrates were present, they were light and consisted mainly of macrophages and/or polymorphonuclear leucocytes. Occasional tumours showed small peripheral collections of lymphocytes and plasma cells. Lymph nodes removed with the excised implant were invariably depleted of lymphocytes and inactive.

*Invasion.*—Appearances suggestive of invasion of surrounding tissues were rare. Occasionally, clumps of tumour cells were present in lymphatics near the implant, one bladder carcinoma was seen to invade muscle and in one ovarian carcinoma deposits were present in the omentum. However, in this last case it is possible that the peritoneum was penetrated during the injection. The finding of clumps of tumour cells in the implant area not continuous with the main mass of growing tumour is not in itself evidence of spread as the injected inoculum is a suspension of tumour fragments which may be distributed fairly widely during injection.

*Relation between growth and tumour type and grade of malignancy*

Tables II–VI shows that a third of all tumours gave good growth and another third survived or grew poorly. The best results were given by melanomata—good growth was obtained with two-thirds of tumours where the recipients survived the first few days. No breast carcinoma

TABLE II.—*Overall Fate of Human Tumours in Immunosuppressed Mice*

| Tumour type | Total | Recipients died | Growth 0 | + | ++ |
|---|---|---|---|---|---|
| Ovarian (see Table III) | 35 | 0 | 7 | 17 | 11 |
| Bladder (see Table IV) | 20 | 0 | 5 | 8 | 7 |
| Melanoma | 16 | 2 | 1 | 4 | 9 |
| Gastrointestinal tract (see Table V) | 12 | 6 | 1 | 2 | 3 |
| Breast | 9 | 1 | 2 | 6 | 0 |
| Uterine carcinoma | 4 | 0 | 0 | 2 | 2 |
| Miscellaneous (see Table VI) | 20 | 3 | 11 | 2 | 4 |
| Total | 116 | 12 | 27 | 41 | 36 |
| (%) | (100) | (10) | (23) | (35) | (31) |

TABLE III.—*Ovarian Tumours: Growth in Immunosuppressed Mice*

| Malignant tumours | Total | Growth 0 | + | ++ |
|---|---|---|---|---|
| Serous adenocarcinoma | 12 | 0 | 6 | 6 |
| Mucinous adenocarcinoma | 6 | 1 | 4 | 1 |
| Poorly differentiated adenocarcinoma | 2 | 0 | 2 | 0 |
| Endometroid carcinoma | 2 | 0 | 1 | 1 |
| Granulosa cell carcinoma | 1 | 0 | 0 | 1 |
| Mesonephroid carcinoma | 1 | 0 | 1 | 0 |
| Dysgerminoma | 1 | 1 | 0 | 0 |
| Lipoidal cell tumour | 1 | 1 | 0 | 0 |
| Endodermal sinus tumour | 1 | 1 | 0 | 0 |
| Teratoma | 1 | 0 | 0 | 1 |
| Total | 28 | 4 | 14 | 10 |
| **Benign tumours** | | | | |
| Fibroma | 2 | 1 | 1 | 0 |
| Mucinous cystadenoma | 2 | 1 | 1 | 0 |
| Serous cystadenoma | 1 | 1 | 0 | 0 |
| Brenner tumour | 1 | 0 | 1 | 0 |
| Teratoma | 1 | 0 | 0 | 1 |
| Total | 7 | 3 | 3 | 1 |

grew well, but most survived or grew poorly for at least 3–7 weeks.

With sarcomata it was difficult to decide whether transplantation was successful for in many cases their structure was not distinguishable with confidence from reactively proliferating host connective tissues. Accordingly, except in the case of one uterine leiomyosarcoma which grew well and showed frequent mitotic figures, we could not positively identify tumour tissue in implants of any of the few sarcomata we examined (Table VI).

There was no evident relation between degree of malignancy and ability to grow in recipient mice. This point is illustrated by the 20 transplanted bladder carcinomata that were graded histologically (Table IV). Similarly, good, poor and no growth occurred indiscriminately among ovarian carcinomata that varied from well differentiated to anaplastic, and 4 out of 7 benign ovarian tumours also grew (Table III).

*Serial transfer*

Serial passage of tumours was attempted in 3 cases only, and succeeded in all. A papillary adenocarcinoma of the ovary that had been growing in mice for 60 days was passaged in secondary recipients for 77 days and in tertiary recipients for 42 days. An ovarian teratoma was passaged after 104 days and the secondary transplants maintained for a further 126 days. A gastric carcinoma transferred after 66 days was maintained for a further 98 days. In all cases tumour was growing well when the experiments

TABLE IV.—*Bladder Tumours: Growth in Immunosuppressed Mice*

| | Total | Growth 0 | + | ++ |
|---|---|---|---|---|
| Transitional cell carcinomata | | | | |
| Grade I | 4 | 0 | 3 | 1 |
| Grade II | 5 | 1 | 3 | 1 |
| Grade III | 3 | 0 | 1 | 2 |
| Grade IV | 7 | 3 | 1 | 3 |
| Squamous cell carcinoma | 1 | 1 | 0 | 0 |
| Total | 20 | 5 | 8 | 7 |

TABLE V.—*Gastrointestinal Tract Tumours: Growth in Immunosuppressed Mice*

| | Total | Recipients died | Growth 0 | Growth + | Growth ++ |
|---|---|---|---|---|---|
| Carcinoma oesophagus | 4 | 3 | 0 | 1 | 0 |
| Carcinoma stomach | 2 | 1 | 0 | 0 | 1 |
| Carcinoma colon | 2 | 1 | 0 | 0 | 1 |
| Adenocarcinoma salivary gland | 1 | 1 | 0 | 0 | 0 |
| Squamous cell carcinoma parotid | 1 | 0 | 0 | 0 | 1 |
| Squamous cell carcinoma oropharynx | 2 | 0 | 1 | 1 | 0 |
| Total | 12 | 6 | 1 | 2 | 3 |

TABLE VI.—*Miscellaneous Tumours: Growth in Immunosuppressed Mice*

| | Total | Recipients died | Growth 0 | Growth + | Growth ++ |
|---|---|---|---|---|---|
| Leiomyosarcoma uterus | 2 | 0 | 1 | 0 | 1 |
| Lymphosarcoma | 2 | 1 | 1 | 0 | 0 |
| Reticulosarcoma | 1 | 0 | 1 | 0 | 0 |
| Medulloblastoma | 2 | 0 | 2 | 0 | 0 |
| Neuroblastoma | 2 | 0 | 2 | 0 | 0 |
| Testicular teratoma (malignant) | 2 | 0 | 0 | 0 | 2 |
| Adenocarcinoma nasal antrum | 1 | 1 | 0 | 0 | 0 |
| Wilm's tumour | 1 | 1 | 0 | 0 | 0 |
| Bronchial carcinoid | 1 | 0 | 1 | 0 | 0 |
| Origin unknown | | | | | |
| Carcinoma, deposit in omentum | 1 | 0 | 0 | 0 | 1 |
| Malignant ascites | 5 | 0 | 3 | 2 | 0 |
| Total | 20 | 3 | 11 | 2 | 4 |

FIG. 1. Tumour 5. Ovarian serous cystadenocarcinoma 17 days after implantation. H. & E. ×450.

FIG. 2. Tumour 5, 17 days after implantation and 3 days after treatment with chlorambucil, 5 mg/kg. Appearances are not significantly different from those of the untreated tumour (Fig. 1). H. & E. ×450.

FIG. 3. Tumour 5, 17 days after implantation and 3 days after treatment with cyclophosphamide, 200 mg/kg. Marked metaphase arrest. H. & E. ×450.

TABLE VII.—*Effect of Chemotherapy on Transplanted Human Tumours*

| Tumour No. | Tumour type | Control growth | Drug | Dose (mg/kg) | Day† given | Day examined | Effect |
|---|---|---|---|---|---|---|---|
| 1 | Ovary, serous cystadenocarcinoma | + | Chlorambucil | 5 | 7 | 21 | None |
| 2 | Ovary, serous cystadenocarcinoma | + | Cyclophosphamide | 100 | 7 | 10 | None |
| 3 | Ovary, serous cystadenocarcinoma | + | Chlorambucil | 5 | 5 | 19 | None |
| 4 | Ovary, serous cystadenocarcinoma | + | Chlorambucil | 5 | 14 | 18 | None |
| | | | Trenimon | 0·2 | 14 | 18 | None |
| 5 | Ovary, serous cystadenocarcinoma | ++ | Cyclophosphamide | 200 | 14 | 17 | Mitotic arrest (Fig. 3) |
| | | | Chlorambucil | 5 | 14 | 17 | None (Fig. 2) |
| 6 | Ovary, serous cystadenocarcinoma | ++ | Cyclophosphamide | 200 | 13 | 16 | None |
| | | | Chlorambucil | 5 | 13 | 16 | None |
| 7 | Ovary, serous cystadenocarcinoma | ++ | Cyclophosphamide | 200 | 22 | 25 | None |
| | | | Trenimon | 0·25 | 22 | 25 | None |
| | | | Actinomycin | 0·375 | 22 | 25 | None |
| 8 | Ovary, serous cystadenocarcinoma | + | Cyclophosphamide | 300 | 14 | 17 | None |
| | | | Chlorambucil | 10 | 14 | 17 | None |
| | | | Thiotepa | 5 | 14 | 17 | None |
| 9 | Ovary, serous cystadenocarcinoma | + | Cyclophosphamide | 300 | 14 | 18 | None |
| | | | Chlorambucil | 15 | 14 | 18 | None |
| | | | 5-Fluorouracil | 100 | 14 | 18 | None |
| 10 | Ovary, mucinous cystadenocarcinoma | ++ | Chlorambucil | 5 | 6 | 20 | None |
| 11 | Ovary, mucinous cystadenocarcinoma | + | Chlorambucil | 5 | 18 | 21 | None |
| 12 | Ovary, mucinous cystadenocarcinoma | ++ | Cyclophosphamide | 100 | 16 | 19 | None |
| 13 | Ovary, mucinous cystadenocarcinoma | + | Cyclophosphamide | 300 | 13 | 17 | None |
| 14 | Ovary, poorly differentiated adenocarcinoma | + | Chlorambucil | 5 | 14 | 18 | None |
| 15 | Ovary, poorly differentiated adenocarcinoma | + | Chlorambucil | 5 | 9 | 16 | None |
| 16 | Ovary, granulosa cell carcinoma | ++ | Chlorambucil | 5 | 6 | 21 | None |
| 17 | Ovary, endometroid carcinoma | ++ | Trenimon | 0·2 | 14 | 17 | None |
| 18 | Ovary, benign teratoma | ++ | Cyclophosphamide | 300 | 21 | 24, 28 | None |
| | | | 5-Fluorouracil | 100 | 21 | 24, 28 | None |
| 19 | Melanoma | + | Cyclophosphamide | 100 | 6 | 20 | None |
| | | | Methotrexate | 1×5 | 6 | 20 | None |
| 20 | Melanoma | ++ | Melphalan | 2·5 | 5 | 19 | Giant cells (Fig. 12) |
| 21 | Melanoma | + | Melphalan | 2·5 | 6 | 18 | None |

| | | | | | | | |
|---|---|---|---|---|---|---|---|
| 22 | Melanoma | + | Melphalan | 2·5 | 14 | 20 | None |
| 23 | Melanoma | + | Melphalan | 2·5 | 14 | 17, 21 | Pyknosis |
| | | | Cyclophosphamide | 100 | 12 | 18 | None |
| 24 | Melanoma | ++ | Melphalan | 2·5 | 8 | 11 | None |
| 25 | Melanoma | ++ | Combined* | — | 14 | 17 | None |
| 26 | Melanoma | ++ | Melphalan | 2·5 | 33 | 35 | None |
| 27 | Melanoma | ++ | Melphalan | 2·5 | 16 | 19 | None |
| 28 | Bladder, transitional cell carcinoma, grade I | + | Methotrexate | 1 × 5 | 5 | 17 | None |
| | | | Cyclophosphamide | 100 | 5 | 17 | None |
| 29 | Bladder, transitional cell carcinoma, grade I | ++ | Methotrexate | 1 × 5 | 5 | 11, 17 | None |
| | | | Melphalan | 2·5 | 5 | 18 | None |
| 30 | Bladder, transitional cell carcinoma, grade I | + | Methotrexate | 1 × 5 | 19 | 26, 30 | None |
| | | | Epodyl | 250 | 23 | 30 | None |
| 31 | Bladder, transitional cell carcinoma, grade II | + | Methotrexate | 1 × 5 | 5 | 20 | None |
| 32 | Bladder, transitional cell carcinoma, grade II | + | Methotrexate | 1 × 5 | 11 | 18, 21 | Squamous metaplasia (Fig. 14) |
| 33 | Bladder, transitional cell carcinoma, grade III | ++ | Methotrexate | 1 × 5 | 6 | 13 | None |
| 34 | Bladder, transitional cell carcinoma, grade III | ++ | Methotrexate | 1 × 5 | 19 | 26, 30 | None |
| 35 | Bladder, transitional cell carcinoma, grade IV | ++ | Methotrexate | 1 × 5 | 12 | 19 | None |
| 36 | Uterus, adenocarcinoma | ++ | Thiotepa | 5 | 14 | 17 | None |
| 37 | Uterus, adenocarcinoma | + | Cyclophosphamide | 300 | 21 | 24, 29 | None |
| 38 | Uterus, adenocarcinoma | + | Cyclophosphamide | 300 | 20 | 24 | None |
| 39 | Uterus, undifferentiated carcinoma | + | Cyclophosphamide | 100 | 19 | 21 | None |
| 40 | Uterus, leiomyosarcoma | ++ | Chlorambucil | 5 | 15 | 22 | None |
| 41 | Testis, malignant teratoma | ++ | Vinblastine | 0·5 | 6 | 21 | None |
| 42 | Parotid, squamous cell carcinoma | ++ | Cyclophosphamide | 100 | 6 | 21 | Marked destruction (Fig. 5) |
| 43 | Omental carcinoma, origin unknown | ++ | Chlorambucil | 5 | 5 | 19 | Marked destruction (Fig, 8, 9) |
| 44 | Ascites, origin unknown | + | Actinomycin | 0·375 | 14 | 18 | None |
| | | | Vinblastine | 0·5 | 14 | 18 | None |

* Cyclophosphamide 187 mg/kg + 5-fluorouracil 156 mg/kg + vincristine 0·31 mg/kg + methotrexate 3 mg/kg × 2.

† Day after implantation that a single dose of drug, or the first dose of a series, was given.

were discontinued. There would therefore seem to be little difficulty in serially transplanting tumours that grow well in the first instance, as noted also by Cobb (1973) and Detre and Gazet (1973).

*Effects of chemotherapy*

In 44 cases implanted mice were treated with the drug or drugs indicated by the clinician as most likely to be used in the patient if chemotherapy proved necessary (Table VII).

It was not possible to assess the effects of chemotherapy by measuring tumour diameter or weighing the excised tumour because the nodules were usually small, grew slowly, contained widely varying amounts of fibrous tissue and could not be distinguished by palpation from nodules of fibrous or necrotic tissue or abscesses. Accordingly, assessment was made by histological examination of randomized coded slides. The results are shown in Table VII.

In the majority of tumours, chemotherapy had no evident effect, the appearance of treated tumours being within the range of variation of that seen in untreated tumours. In 3 cases, an unequivocal effect was seen:

(1) *Tumour 5.*—This was a poorly differentiated serous cystadenocarcinoma of the ovary with frequent mitotic figures, which grew well in recipient mice (Fig. 1). Three days after treatment with cyclophosphamide, 200 mg/kg body weight, there was a conspicuous metaphase arrest (Fig. 3). In contrast, treatment with chlorambucil, 5 mg/kg, had no discernible effect (Fig. 2).

(2) *Tumour 42.*—This was a squamous cell carcinoma of the parotid which grew well in recipient mice, showing a typical structure and producing keratin (Fig. 4). Two weeks after giving cyclophosphamide, 100 mg/kg, only small fragments of tumour remained, consisting largely of keratinized cells, and surrounded by fibrous tissue infiltrated with polymorphs (Fig. 5).

(3) *Tumour 43.*—This was an omental metastasis of a poorly differentiated, pleomorphic carcinoma, with frequent mitoses. It was of unknown origin (Fig. 6). Two weeks after giving chlorambucil, 5 mg/kg, the only evidence of residual tumour was a few giant cells with vacuolated cytoplasm that were probably damaged tumour cells (Fig. 8). The remainder of the excised nodules consisted of fibrous or necrotic tissue infiltrated with polymorphonuclear leucocytes and plasma cells (Fig. 9). Tumours in untreated mice continued to grow well (Fig. 7).

In 3 other cases lesser effects were seen. These occurred in all or the majority of treated mice and not in the controls, and could reasonably be attributed to drug action. These changes were: (1) excessive numbers of tumour giant cells in a melanoma (tumour 20) treated with melphalan (Fig. 11, 12); (2) increased numbers of pyknotic nuclei in a melanoma (tumour 23) after treatment with melphalan but not with cyclophosphamide; and (3) squamous metaplasia in a bladder carcinoma (tumour 32) after treatment with methotrexate (Fig. 13, 14). The significance of such changes is not clear and they must be regarded as equivocal until the subsequent fate of tumours that show them is known.

In a melanoma (tumour 20) that consisted of structurally different clones, these appeared to differ in their drug susceptibility, *i.e.* a drug effect was seen in one clonal type, but not in an adjacent clone of different types (Fig. 11, 12).

### DISCUSSION

This investigation shows that human tumours can be grown with fair regularity in thymectomized, irradiated, ALS-treated mice. In our hands two-thirds of all tumours survived or grew, sometimes for months. If tumours are excluded the recipients of which died within a few

FIG. 4. Tumour 42. Squamous cell carcinoma of the parotid 21 days after implantation. H. & E. ×108.

FIG. 5. Tumour 42, 21 days after implantation and 15 days after treatment with cyclophosphamide, 100 mg/kg. Tumour tissue has largely disappeared and has been replaced by fibrous tissue infiltrated with polymorphs. H. & E. ×108.

FIG. 6. Tumour 43, carcinomatous secondary in omentum, origin unknown. Original tumour. H. & E. ×108.

FIG. 7. Tumour 43, 19 days after implantation. Note solid structure compared with primary tumour (Fig. 6). H. & E. ×108.

FIG. 8. Tumour 43, 19 days after implantation and 14 days after treatment with chlorambucil, 5 mg/kg. A small nodule of tumour giant cells, surrounded by fibrous tissue infiltrated with polymorphs and plasma cells, is the only remnant of the tumour. H. & E. ×45.

FIG. 9.—As Fig. 8, showing complete destruction of the remainder of the tumour. H. & E. ×108.

FIG. 10.—Tumour 20, melanoma, showing primary tumour. H. & E. ×108.

days, presumably from infection, about three-quarters of the tumours (and 60% of implants) survived or grew. These results are appreciably better than those obtained on primary transplantation to the cheek pouch of the cortisone treated hamster, where some 16–45% of tumours survived or grew (Handler, Davies and Sommers, 1956; Patterson, Patterson and Chute, 1957; Patterson, 1968; Williams, Evans and Blamey, 1971) or to thymectomized, ALS-treated, unirradiated mice, in which about a quarter do so (Phillips and Gazet, 1970). Comparable results (about two-thirds successful transplants) were obtained by Castro (1972) who implanted tumours beneath the kidney capsule of thymectomized, irradiated, bone marrow-reconstituted mice. Cobb (1972) transplanted human tumours either subcutaneously or into the cheek pouch of thymectomized, ALS-treated hamsters. About half the subcutaneously implanted tumours and 80% of those placed in the cheek pouch grew locally. However, the proportion of successful implants was low so that when growth was obtained it was usually observed in only 1 or 2 recipients in an average group of 5. It was interesting that the majority of tumours metastasized to the lungs, even when they did not grow at the implantation site. It is unlikely that the optimum conditions for growing human tumours in xenogeneic hosts have been found yet, and systematic comparison of various species and host treatments is required. Nude mice may prove useful for this purpose (Rygaard and Povlsen, 1969; Povlsen and Rygaard, 1971; Povlsen *et al.*, 1973), particularly if problems of supply and short life span can be overcome.

In successfully transplanted tumours the characteristic histological appearances are generally retained to a striking extent, as has been noted by many other workers (Handler *et al.*, 1956; Patterson, 1968; Williams *et al.*, 1971; Castro, 1972; Cobb, 1972, 1973). This suggests that many of the biochemical features

FIG. 11.—Tumour 20, 19 days after implantation, showing structurally different clones of tumour cells. H. & E. ×180.

FIG. 12.—Tumour 20, 19 days after implantation and 14 days after treatment with melphalan, 2·5 mg/kg, showing formation of tumour giant cells in one clone and no evident change in the other. H. & E. ×180.

FIG. 13.—Tumour 32, transitional cell carcinoma of the bladder 21 days after implantation. H. & E. ×108.

FIG. 14.—Tumour 32, 21 days after implantation and 6 days after end of a course of methotrexate, 1 mg/kg/day × 5, showing squamous metaplasia and increased infiltration with plasma cells and macro- [illegible]

of the original tumour are also retained, for a marked change in these would not be consistent with retention of morphological characteristics and such differentiated functions as production of mucin and keratin. It is also relevant that continuing endocrine function is shown by human choriocarcinoma and parathyroid adenoma tissue transplanted to immunosuppressed hamsters, rats and mice (Hertz, 1959; Castro and Zanelli, 1973), and continued production of immunoglobulin and virus antigen by Burkitt's lymphoma tissue transplanted to nude mice (Povlsen *et al.*, 1973). These findings encourage the hope that the drug sensitivity of the original tumour is also retained to a useful extent, a supposition supported by our finding that, when a rat tumour of known drug sensitivity was grown in immunosuppressed mice, the rank order of effectiveness of 4 drugs was identical in the species of origin and in the xenogeneic host (Sheard *et al.*, 1971).

This model therefore may be a suitable one in which to investigate the drug sensitivity of individual human tumours. However, caution is required in view of the finding of Cobb (1973) and Detre and Gazet (1973) that the frequency of mitoses in human tumours examined some months after transplantation to immunosuppressed mice was usually higher than in the primary tumour. It is not known whether this was due to these relatively small tumour masses being better vascularized than the larger primaries from which they were derived, or whether selection of rapidly growing cells had occurred during the 3–8 months sojourn in the xenogeneic host.

There are practical difficulties in determining the drug sensitivity of tumour transplants. Because of the varying composition of the implant nodules, methods of assessment that are not histological must always be accompanied by careful histological examination, but this is a relatively crude method of evaluation which may fail to reveal some important types of drug-induced damage. On the other hand, changes may be seen, the significance of which is difficult to assess and which may be unimportant in the long run. Because of these difficulties, and the additional problem of sampling error, we adopted stringent criteria in evaluating the changes we saw. In only 3 of the 44 transplanted tumours we treated did we feel confident in describing the damage as severe, that is, we would have expected a marked objective response to occur if such changes had been seen in a treated patient. Another 3 tumours showed lesser damage, the significance of which could not be assessed in the absence of serial studies.

In practice, it is the difficulty of determining the effect of the drug on the tumour in the patient rather than in the recipient mouse, that is likely to present the greater problem. In only a minority of tumours in patients is direct measurement possible during therapy, repeated biopsies are rarely justifiable and in most patients surgery and/or radiotherapy are also used and their timing often makes it impossible to assess the effect of chemotherapy alone.

Two findings call for particular comment. First, tumour 5 showed marked damage to the mitotic apparatus after treatment with cyclophosphamide but was apparently unaffected by chlorambucil (Fig. 2, 3), and tumour 23 showed increased numbers of pyknotic nuclei after melphalan but not after cyclophosphamide. Second, some tumour implants showed histologically different clonal types and in at least one of these (tumour 20) the clones appeared to differ in sensitivity to melphalan, at least as shown by structural changes after drug administration (Fig. 11, 12). These differences in effect between 2 agents of the same class acting on the same tumour, and between different clones of the one tumour, suggest that this model shows substantial discrimination and supports the belief that the changes observed are not therapeutically irrelevant or nonspecific but do indeed reflect tumour drug sensitivity.

Other workers have also shown that human tumours growing in xenogeneic hosts may be damaged by chemotherapeutic agents. These studies have mainly involved later generations of repeatedly passaged tumours which have been shown to be sensitive to a variety of agents (Hertz, 1960; Friedell, Sherman and Sommers, 1961; Kaufman and Lichtenauer, 1968; Adams *et al.*, 1972). However, the extent to which such repeatedly transplanted tumours retain their initial drug sensitivity is unknown. Smith (1969) found that first generation implants of human tumours in the hamster cheek pouch varied in sensitivity to methotrexate and nitrogen mustard, and suggested that this variation implied varying drug sensitivities in the original tumours. However, as the latter sensitivities were not known, such a relationship could not be ascertained.

The only study so far that has been addressed directly to the problem of comparing the drug sensitivity of a transplanted tumour with that of the primary is that of Burt *et al.* (1966), who describe a patient with a carcinoma of the bladder that was sensitive to 5-fluorouracil and x-rays and resistant to vinblastine and that retained these characteristics when grown in immunosuppressed hamsters. This correspondence in a single patient is encouraging, but it is evident that any assay intended for use in determining the drug sensitivity of individual human tumours should be validated in a statistically significant manner in a reasonable number of patients.

Two approaches to validation of such an assay may be considered. The assay would be validated if, for example, the results of chemotherapy in patients receiving drugs indicated by the assay were significantly better than those in comparable patients receiving drugs according to currently accepted criteria and without assay. This approach appears simple in theory but would be exceedingly difficult in practice. There is a wide variation in the results of chemotherapy obtained by different clinical units and from one period to another in the same unit, due largely to uncontrollable factors such as acquisition of expertise and the great inherent variability of cancer patients and their responses to treatment. It is therefore not warrantable simply to compare results obtained before the assay is adopted by a particular unit with those obtained afterwards, nor to compare the results obtained by units using the assay with those of units not using it. Unless the improvement in results following adoption of the assay was so great that the role of such uncontrolled variables could be discounted, a properly designed prospective trial would be needed, in which the 2 groups of patients were randomized from the same intake and treated contemporaneously by the same personnel, to show whether use of the assay influenced the results of chemotherapy. Such a trial would be a formidable undertaking and, for ethical reasons, it could only involve clinicians who did not believe that a deliberate choice of drug based on personal experience and knowledge was likely to be better than a choice based on an unvalidated laboratory assay.

These difficulties are probably insuperable and consequently the temptation to allow such laboratory assays to influence the choice of drug before they are thoroughly validated is not always resisted. Unfortunately, the incorporation of an unvalidated method into regular practice can make it very difficult later to carry out the sort of unbiased investigation that alone can determine the usefulness or otherwise of the method.

We therefore propose an alternative approach, briefly outlined earlier (Berenbaum and Sheard, 1972) based on the following considerations. On the whole, clinicians tend to treat most patients with a particular type of tumour with one or a few drugs shown by experience to be beneficial in a useful proportion of cases. Tumours of a particular type generally show a spectrum of sensitivity to any one drug and, in any individual patient, the drug may produce anything from complete remission to no benefit whatsoever. The effects of treating human tumours transplanted to mice also vary, as we have shown here, from severe damage to none. In principle, it should not be difficult to determine whether the relatively few tumours that are markedly damaged in the mouse show a statistically satisfactory correspondence with the few where the patient has benefited markedly from the same drug. If this proves to be the case, the assay will be validated. Accordingly, the choice of drugs to be used in individual patients is left entirely to the clinician and the effects of these drugs only are assayed on the same tumours. The results of assaying the effects of drugs that the patient does not receive are irrelevant, for such assays cannot be validated. However, in those cases in which no choice of drug has been made at the time of assay, the 2 or 3 drugs most likely to be used are tested.

If the assay is validated it can then be used predictively, in the first instance to detect patients whose tumours are likely to show substantial regression under treatment with the drug in question. If it appears that the assay is valid for a variety of drugs, the logical next step would be to modify it to find which drug out of those available would be most suitable for use in an individual patient. There are no ethical difficulties in this approach and it can be effected concomitantly with orthodox treatment.

We are grateful to the Cancer Research Campaign, the Medical Research Council, the Nuffield Foundation and the Leukaemia Research Fund for financial support, to Dr J. A. Double and Mr B. Trefty for assistance, and to the surgeons and pathologists of the following hospitals for generously supplying us with tumours: St Mary's Hospital Group, St Bartholomew's Hospital Group, St Thomas' Hospital, the Royal Marsden Hospital, the Hospital for Sick Children, Great Ormond St, the Hammersmith Hospital, Central Middlesex Hospital, St James' Hospital, Balham and Addenbrooke's Hospital, Cambridge.

### REFERENCES

Adams, R. A., Flowers, A., Sundeen, R. & Merk, L. P. (1972) Chemotherapy and Immunotherapy of Three Human Lymphomas Serially Transplantable in the Neonatal Syrian Hamster. *Cancer, N.Y.*, **29**, 524.

Berenbaum, M. C. & Sheard, C. E. (1972) Testing Anti-cancer Drugs. *Lancet*, i, 1116.

Burt, F. B., Pavone-Macaluso, M., Horns, J. W. & Kaufman, J. J. (1966) Heterotransplantation of Bladder Cancer in the Hamster Cheek Pouch: *in vivo* Testing of Cancer Chemotherapeutic Agents. *J. Urol.*, **95**, 51.

Castro, J. E. (1972) Human Tumours Grown in Mice. *Nature, New Biol.*, **239**, 83.

Castro, J. E. & Zanelli, J. M. (1973) Human Parathyroid Adenomata Maintained in Immunosuppressed Mice. *Nature, New Biol.*, **245**, 51.

Cobb, L. M. (1972) Metastatic Spread of Human Tumour Implanted into Thymectomized Antilymphocytic Serum Treated Hamsters. *Br. J. Cancer*, **26**, 183.

Cobb, L. M. (1973) The Behaviour of Carcinoma of the Large Bowel in Man following Transplantation into Immune Deprived Mice. *Br. J. Cancer*, **28**, 400.

Detre, S. I. & Gazet, J. C. (1973) Transplantation of Human Tumour to Immune Deprived Mice Treated with Anti-thymocyte Serum. *Br. J. Cancer*, **28**, 412.

Editorial (1972) Testing Anti-cancer Drugs. *Lancet*, i, 827.

Friedell, G. H., Sherman, J. D. & Sommers, S. C. (1961) Growth Curves of Human Cancer Transplants during Experimental Chemotherapy. *Cancer, N.Y.*, **14**, 1117.

Handler, A. M., Davies, S. & Sommers, S. C. (1956) Heterotransplantation Experiments with Human Cancers. *Cancer Res.*, **16**, 32.

Hertz, R. (1959) Choriocarcinoma of Women Maintained in Serial Passage in Hamster and Rat. *Proc. Soc. exp. Biol. Med.*, **102**, 77.

Hertz, R. (1960) Suppression of Human Choriocarcinoma Maintained in the Hamster Cheek Pouch by Extracts and Alkaloids of *Vinca rosea*. *Proc. Soc. exp. Biol. Med.*, **105**, 281.

Kaufman, J. J. & Lichtenauer, P. (1968) Cancer of Human Bladder. Responses of Tumour Xenografts to Chemotherapy and Radiotherapy. *Cancer, N.Y.*, **21**, 1.

Levey, R. H. & Medawar, P. B. (1966) Some Experiments on the Action of Antilymphoid Antisera. *Ann. N.Y. Acad. Sci.*, **129**, 164.

Patterson, W. B. (1968) Transplantation of Human Cancers to Hamster Cheek Pouches. *Cancer Res.*, **28**, 1637.

PATTERSON, W. B., PATTERSON, H. R. & CHUTE, R. N. (1957) Transplantable Human Cancers. *Cancer, N.Y.*, **10**, 1281.

PHILLIPS, B. & GAZET, J. C. (1970) Transplantation of Primary Explants of Human Tumour to Mice Treated with Antilymphocyte Serum. *Br. J. Cancer*, **24**, 92.

POVLSEN, C. O., FIALKOW, P. J., KLEIN, E., KLEIN, G., RYGAARD, J. & WIENER, F. (1973) Growth and Antigenic Properties of a Biopsy-derived Burkitt's Lymphoma in Thymusless (Nude) Mice. *Int. J. Cancer*, **11**, 30.

POVLSEN, C. O. & RYGAARD, J. (1971) Heterotransplantation of Human Adenocarcinomas of the Colon and Rectum to the Mouse Mutant Nude. A Study of Nine Consecutive Transplantations. *Acta path. microbiol. scand.*, **79**, 159.

RYGAARD, J. & POVLSEN, C. O. (1969) Heterotransplantation of a Human Malignant Tumour to " Nude " Mice. *Acta path. microbiol. scand.*, **77**, 758.

SHEARD, C. E., DOUBLE, J. A. & BERENBAUM, M. C. (1971) The Sensitivity to Chemotherapeutic Agents of a Rat Tumour Grown in Immunosuppressed Mice. *Br. J. Cancer*, **25**, 838.

SMITH, G. M. R. (1969) The Effect of Cytotoxic Agents on Human Tumours Transplanted to the Hamster Cheek Pouch. *Br. J. Cancer*, **23**, 78.

WILLIAMS, D. E., EVANS, D. M. D. & BLAMEY, R. W. (1971) The Primary Implantation of Human Tumours to the Hamster Cheek Pouch. *Br. J. Cancer*, **25**, 533.